# Dewatering Green Sapwood Using Carbon Dioxide Undergoing Cyclical Phase Change between Supercritical Fluid and Gas

**DOI:** 10.3390/molecules25225367

**Published:** 2020-11-17

**Authors:** Robert A. Franich, Roger Meder, Volker C. Behr

**Affiliations:** 1 Chemipreneur Limited, Rotorua 3010, New Zealand; 2 Meder Consulting, Queensland QLD 4017, Australia; roger@mederconsulting.com; 3 Experimental Physics 5, University of Würzburg, 97074 Würzburg, Germany; behr@physik.uni-wuerzburg.de

**Keywords:** supercritical CO_2_, phase-change, sapwood, dewatering, physical chemistry, nuclear magnetic resonance spectroscopy, magnetic resonance imaging

## Abstract

Conventional kiln drying of wood operates by the evaporation of water at elevated temperature. In the initial stage of drying, mobile water in the wood cell lumen evaporates. More slowly, water bound in the wood cell walls evaporates, requiring the breaking of hydrogen bonds between water molecules and cellulose and hemicellulose polymers in the cell wall. An alternative for wood kiln drying is a patented process for green wood dewatering through the molecular interaction of supercritical carbon dioxide with water of wood cell sap. When the system pressure is reduced to below the critical point, phase change from supercritical fluid to gas occurs with a consequent large change in CO_2_ volume. This results in the efficient, rapid, mechanical expulsion of liquid sap from wood. The end-point of this cyclical phase-change process is wood dewatered to the cell wall fibre saturation point. This paper describes dewatering over a range of green wood specimen sizes, from laboratory physical chemistry studies to pilot-plant trials. Magnetic resonance imaging and nuclear magnetic resonance spectroscopy were applied to study the fundamental mechanisms of the process, which were contrasted with similar studies of conventional thermal wood drying. In conclusion, opportunities and impediments towards the commercialisation of the green wood dewatering process are discussed.

## 1. Introduction

### 1.1. Conventional Processes for Drying Green Industrial Wood

Wood freshly sawn from a log, often referred to as “green” wood, usually has a high sapwood moisture content, particularly in softwood species such as pines. A well-known example of an industrial wood grown in high-production plantation forests in New Zealand is radiata pine (*Pinus radiata* D. Don), the green sapwood moisture content of which is typically ca. 150–200% based on the wood material oven-dry weight [1].

The conversion of green wood to utility wood requires removing the water to a point where the wood material has dimensional and conformational stability and is non-perishable. The moisture content of dry wood (<ca. 20%) is sufficiently low so that microorganisms such as moulds and fungi cannot grow and deteriorate the wood. Removing water from wood cells is carried out commercially using several methods, the simplest by simply stacking the wood with the boards separated to allow passive natural air flow at ambient temperature while the wood stack is kept protected from the weather. Practiced for many centuries, this is often referred to as wood “seasoning”, which is a process that can be enhanced by use of industrial dehumidifiers. While gentle on the wood material, seasoning is considered too slow for high-volume industrial wood production to meet demand.

Higher throughput can be achieved by the application of heat to stacked green wood, which is a process conducted on large industrial scales in drying kilns [1]. In its simplest form, kiln drying is carried out at atmospheric pressure with fan-circulated hot air [2] at temperatures ranging from 70 up to 100–120 °C or higher, with the exhaust from the kiln, comprising steam and volatile compounds derived from thermally modified wood material [3] discharged into the local airspace. Advanced methods of heating the wood cell water to generate water vapour internally include using microwave energy [4] or radiofrequency [5]. 

All these methods require the phase change of cell water from liquid to vapour, which is an energy-demanding process requiring the breaking of hydrogen bonds between water molecules or between water molecules and wood cell wall polymers (cellulose and hemicelluloses) [2]. Within the structure of wood undergoing drying at elevated temperature, water moves between the tracheid cells via interconnecting pits [2]. Using magnetic resonance imaging (MRI), it also has been shown that during kiln drying, cell water moves anisotropically within the earlywood (the spring-wood located between the tree annual rings, anatomically known as “latewood”) of sapwood towards the drying surface [6]. 

A consequence of kiln-drying industrial softwood, such as from radiata pine, is the irreversible closure of the cell wall pits [2], the apertures between cell walls that allow movement of water within the stem of the living tree [7]. The pressure differential generated between adjacent cells during active drying snaps the pits shut, which is an irreversible process known as “pit aspiration” [2]. The ensuing internal moisture gradients generated can also result in distortion of the wood bulk material [8,9], which in commercial practice is minimised by placing massive weights on the wood stack. High-temperature drying may also result in a degree of physical collapse of the wood cell walls, resulting in lowered wood strength. Drying may also promote molecular changes leading to formation of “kiln brown stain” [10], which is exhibited as dark-brown patches revealed during the planing and finishing of the dried wood, thereby compromising wood product quality and value. 

An issue for communities where wood drying kilns are located close to habitation is the often the “nuisance odour” of cooked wood. Kiln emissions from kiln drying of radiata pine wood have been shown to contain the monoterpenes α- and β-pinene as well as toxic molecules such as methanol, formaldehyde, acetaldehyde, furfural, and guaiacol [3]. These compounds arise from the thermal breakdown of the wood cell wall polymers, cellulose, hemicellulose, and lignin.

### 1.2. New Approaches to Industrial Wood Manufacture Using Dewatering Processes

In order to devise a process to rival convention, in which the problems described above might be circumvented, conceptual models whereby the known biophysics and chemistry of cell water movement and hydrogen bonding have been developed to address the fundamental issue of the energy-demanding phase change of cell water from liquid to vapour during wood drying. 

Novel approaches to removing water from green wood have focussed on dewatering using mechanical rather than thermal processes. Compression-rolling of green wood squeezed water from the cells and increased the density and surface hardness of the wood material once dried, but it also reduced the wood material volume (the measure by which wood material is usually sold, e.g., $/m^3^) and destroyed the cell wall structure [11]. Another method applied centrifugal force to high-moisture-containing wood, accelerating the cell-wall water sufficiently to overcome the internal forces between cell water and cell lumens and capillaries, enabling dewatering [12]. 

One promising green wood dewatering process used cycles of compressed air, in which an “incubation” stage, where air molecules were forced into wood cell water under high pressure where they dissolved, before a second, decompression stage, caused dissolution of the air. The subsequent air volume expansion resulted in cell water being forced through the wood structure and out from the wood surface [13]. The “incubation–decompression” cycle was applied 50 or more times in order to dewater wood sufficiently so as to remove all the cell lumen water, leaving only the water bound in the wood cell walls, i.e., wood at the fibre-saturation point. Importantly, in spite of the green wood material having been subjected to many cycles of high pressure and decompression, the cell structure and wood anatomy was unchanged from that of the fresh, green state. A practical benefit of the compressed air “incubation–decompression” process was the elimination of kiln brown-stain [14]. This process, resulting in improved quality of the output wood material, seemed a good candidate process to rival conventional drying.

### 1.3. Theory for a Supercritical Carbon Dioxide-to-Gas Phase-Change Process for Green Wood Dewatering

The application of supercritical carbon dioxide for processing wet materials, such as green wood, is less well known than the numerous applications to dry materials [15]. The solubility of carbon dioxide in bulk water increases with pressure, e.g., for water at 50 °C and at 1 MPa gas pressure, the mole fraction of carbon dioxide in water is 0.00222; at 3 MPa gas pressure, it is 0.00608; at the critical pressure (7.4 MPa), it is 0.01267; and at approximately 17 MPa supercritical fluid pressure, it is 0.01993, whereas, at a constant pressure, the solubility of carbon dioxide in water decreases with increase in temperature [16]. The diffusion of water into the supercritical carbon dioxide phase creates a separate phase, *sc*CO_2_ + H_2_O as a result of the molecular interaction of carbon dioxide and water with a water mole fraction at saturation 0.0011 and critical temperature 31.424 °C [17]. 

Therefore, substituting carbon dioxide for air in an “incubation–decompression” process would potentially reduce the number of cycles required to dewater green wood to the fibre saturation point. To achieve the smallest number of “incubation–decompression” cycles in the fastest time, the highest practicable pressure would be required to enable supercritical carbon dioxide to maximally penetrate the cell water of green wood material. By keeping the process temperature as low as practicable, while keeping the temperature above the critical temperature, 31.1 °C, the maximum solubility of carbon dioxide in the wood cell water would be achieved. The benefit of these conditions is the delivery of a greater amount of carbon dioxide molecules into the wood cell water by using the most dense supercritical fluid phase. Therefore, the generation of supercritical carbon dioxide is a practical means to achieve this. 

Another variable that was considered for the “incubation–decompression” cyclical process was the time the green wood was held at the supercritical carbon dioxide maximum pressure to allow for sufficient diffusion into the cell water to take place. The diffusion coefficient for the carbon dioxide–water system increases with the increase in temperature [18]. In designing experiments to test the hypothesis that the application of supercritical carbon dioxide to green wood would affect dewatering at a faster rate than when using air, several variables were identified. Key variables that could affect the process speed, and therefore efficiency, included pressure, temperature, and hold time at maximum pressure and at the low-pressure decompression stage of the process. Lowering the pressure below the carbon dioxide critical pressure, 7.4 MPa, would also result in phase change of the carbon dioxide from supercritical fluid to gas. There were evidently competing influences of lower temperature for maximising the carbon dioxide solubility and higher temperature for maximising the diffusivity in wood cell water, which would affect the dewatering efficiency.

## 2. Review of Science Studies on Dewatering Green Wood Using the Supercritical Carbon Dioxide-to-Gas Phase-Change Process

### 2.1. Laboratory Experiments to Study Dewatering of Radiata Pine Sapwood Specimens

**Figure 1:**
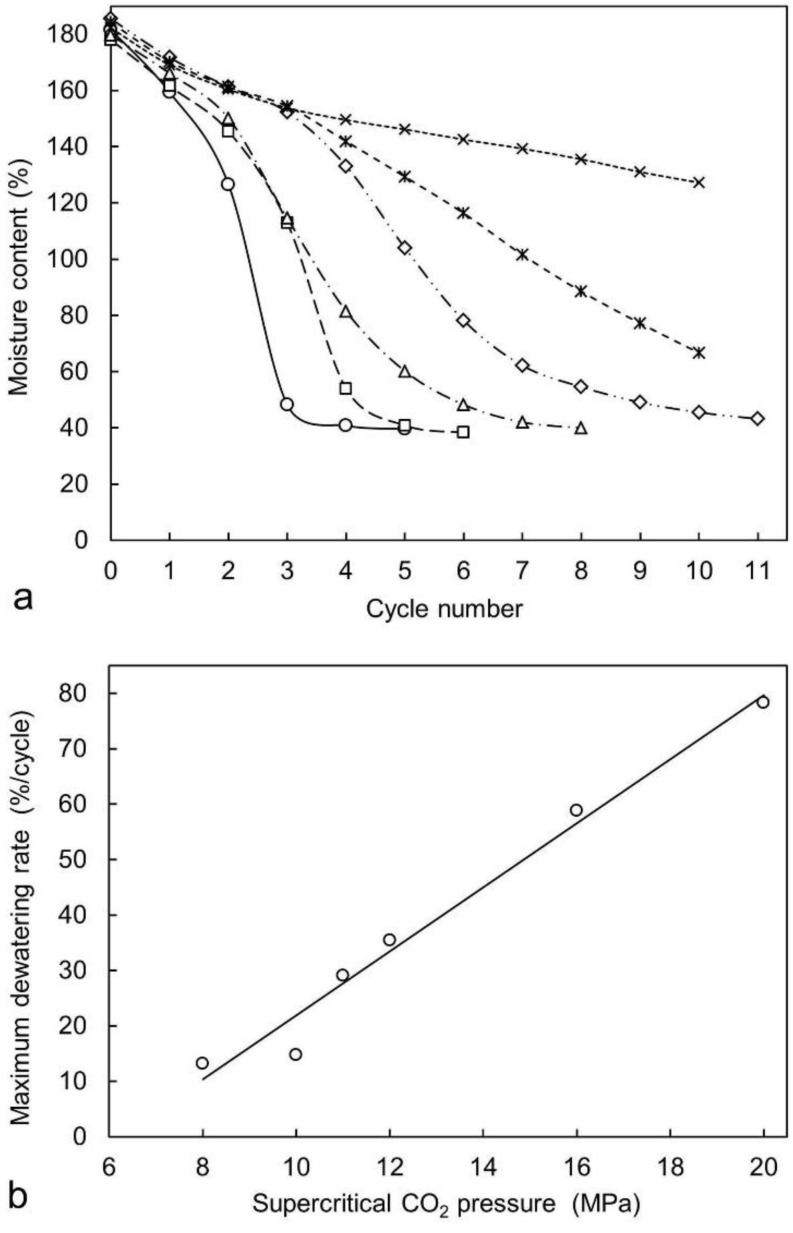
(**a**), Supercritical CO_2_ dewatering curves for radiata pine sapwood specimens with a hold time of 2 min, vessel temperature of 47 °C, and maximum vessel pressures of 8 MPa (crosses), 10 MPa (stars), 11 MPa (diamonds), 12 MPa (triangles), 16 MPa (squares), and 20 MPa (circles) and (**b**), the maximum dewatering rate, calculated from (**a**), as a function of the maximum supercritical carbon dioxide pressure. The line shows a linear least-squares fit [19] (with permission from Elsevier).

Experiments to test the dewatering theory used small green wood specimens, 18 mm square cross-section and 100 mm long, the length dimension being parallel with the axis of the log from which the specimens were sampled. The specimens had an initial moisture content of 180% based on the specimen dry weight. The stainless steel, high pressure, thermally-jacketed vessel used had a top inlet for introducing supercritical carbon dioxide and a lower outlet for draining water and allowing the exit of gaseous carbon dioxide. Supercritical carbon dioxide was generated using a liquid delivery tank, a high-pressure pump, and a water bath at the set-point temperature for the experiments [19,20]. The result of testing the pressure variable vs. the maximum dewatering rate is shown in Figure 1b, which showed a good linear relationship between the dewatering rate and maximum applied supercritical carbon dioxide pressure.

Green wood specimens sampled from the same source were oven-dried at 100 °C to compare the rate of drying from green to 40% moisture content with the rate of dewatering using 20 MPa highest pressure, as shown in Figure 1. For comparable wood specimens starting at comparable green moisture content, the dewatering process, which concluded at 40% moisture content, close to the wood material fibre-saturation point, was approximately seven times faster than oven-drying from green to 40% moisture content. Heating wood dewatered to 40% moisture content at 100 °C produced a drying curve identical with that of wood specimens at 40% moisture content that had been oven-dried from green, indicating that the energy required to evaporate water from wood cell walls was the same regardless of how the water content of the wood specimens had initially been reduced from green to 40% moisture content. 

The maximum dewatering rate at 20 MPa was not significantly increased when the maximum pressure was increased to 40 MPa. There was only a minimal effect of exact temperatures of 38, 47, and 58 °C on the dewatering rate when using 20 MPa as the maximum pressure [19]. Attempts to dewater green wood specimens at a set-point temperature below 38 °C (and above 32 °C to ensure the supercritical carbon dioxide phase was maintained) often resulted in the specimen freezing when the pressure was lowered. Ice formation inside the specimen was a result of the significant reduction in an internal specimen temperature when the phase changed from supercritical fluid to gas [20]. Occasionally, as carbon dioxide gas expanded against ice, the specimen fractured in the longitudinal direction. To guard against the formation of ice during the decompression state and against the wood specimens fracturing, all experiments were carried out at a set-point of 50 °C [19,20]. 

There was a significant effect of the hold time at maximum pressure, with the linear relationship of maximum dewatering rate and square root of the hold time indicating a diffusion-controlled process [19]. Attempts to achieve dewatering at 20 MPa with a long hold time (60 min) and a single cycle did not dewater the wood specimen any more than a single cycle with a hold time of 2 min, which suggests that the diffusion of supercritical carbon dioxide into green wood was sufficiently fast to rapidly achieve a saturated solution in cell water at the maximum pressure of each cycle. Each dewatering cycle, shown in Figure 1, resulted in a reduced moisture content, creating more “empty” volume within the wood specimen for the entry of supercritical carbon dioxide for the next cycle, thereby accelerating the rate of dewatering by the end of cycle 3, as shown in Figure 1.

Experiments carried out with larger dimension equipment and specimen size (up to 35 mm square cross-section and 200 mm long) showed that larger specimens dewatered at a rate faster than did the smaller, indicating that longer tortuous pathways for carbon dioxide gas to expand within the larger wood specimens resulted in pushing out a greater cell water volume per cycle [20]. The moisture content, ca. 40%, at the end of the dewatering cycles was independent of specimen dimensions, and specimen dimensions at 40% moisture content were similar to those of the specimen when green. Pre-heating the green wood specimens to a temperature to match that of the thermally jacketed vessel also increased the dewatering rate as a result of the increased rate of diffusion of supercritical carbon dioxide into the cell water [20].

Application of the process whereby the phase of carbon dioxide changed from supercritical fluid to gas efficiently dewatered green radiata pine sapwood and indicated advantages of speed and dried wood material quality. 

### 2.2. Laboratory Experiments Dewatering Wood Specimens Sampled from Hardwood Timber Species and Softwood Species Other Than Radiata Pine

The use of radiata pine sapwood as a model wood material to study the dewatering process was fortuitous, but also relevant, as radiata pine is a major industrial forestry wood species grown in New Zealand and several other countries, e.g., Australia, Chile, and Spain. To examine the applicability of the supercritical carbon dioxide phase-change dewatering process to wood specimens derived from other timber species, twenty-two hardwood and softwood timber species were committed to the dewatering process described above [21]. (Hardwood and softwood species are described as such because of having different cell wall lignin chemistry, not because of the actual hardness of the wood material itself, e.g., balsa wood (*Ochroma pyramidale*), a very low-density and surface-soft wood, is technically a hardwood). 

In addition to the different cell wall lignin chemistry, hardwood species have wood anatomy different from that of softwoods, such as radiata pine, particularly the inclusion of large water-conducting vessels in the hardwood structure. Among the hardwood species investigated using the supercritical carbon dioxide to gas phase change dewatering process, [21], there was highly variable green moisture content, and, from the results, the dewatering efficiency of the twenty-two timber species studied could be grouped into high efficiency (>90%), medium efficiency, (50–90%) and low efficiency (<30%) dewatering [21]. A small group of four softwood species, including radiata pine, underwent dewatering at the highest efficiency, while a larger group of generally hardwood species were grouped into the medium and low dewatering efficiency category. Two specimens, *Nothofagus fusca* (Beech) and *Thuja plicata* (Thuja), appeared to be impermeable to supercritical carbon dioxide and underwent dewatering poorly, as explained by the high polyphenol and resin extractive contents blocking the intercellular connection pathway of the pits, restricting supercritical carbon dioxide ingress [21].

Among the hardwood timbers utilised industrially, the wood from *Eucalyptus* species is valued for its strength and natural durability. However, the direct kiln drying of green eucalypt wood often results in bulk material distortion and volumetric collapse, as explained by the high internal water tension within the very small wood pores [22]. Importantly, at the end of the dewatering process applied to these collapse-prone timber specimens, all showed no collapse, implying that the cell walls remained fully hydrated after the cell lumens had been emptied of water. One benefit of applying the dewatering process to the hardwood specimens was less distortion on final kiln drying after a dewatering step compared with the degree of distortion seen after kiln drying from green alone [21]. 

In-depth studies of combining dewatering sequences and a final drying step using temperatures typical of conventional kiln drying of industrially important *Eucalyptus* species showed significant advantage in speed of conversion from green to dry compared with current commercial practice and in the final dry material quality [23,24,25]. Whereas carbon dioxide supercritical fluid-to-gas phase change dewatering was straightforward when applied to radiata pine and some other softwoods, application of the process to difficult-to-dry timbers such as the eucalypts might have greater commercial impact by minimising product quality loss, which results from material distortion and volumetric collapse when green eucalypt wood is conventionally kiln dried. 

### 2.3. Dewatering Radiata Pine Sapwood Using Industrial-Scale Specimens

**Figure 2:**
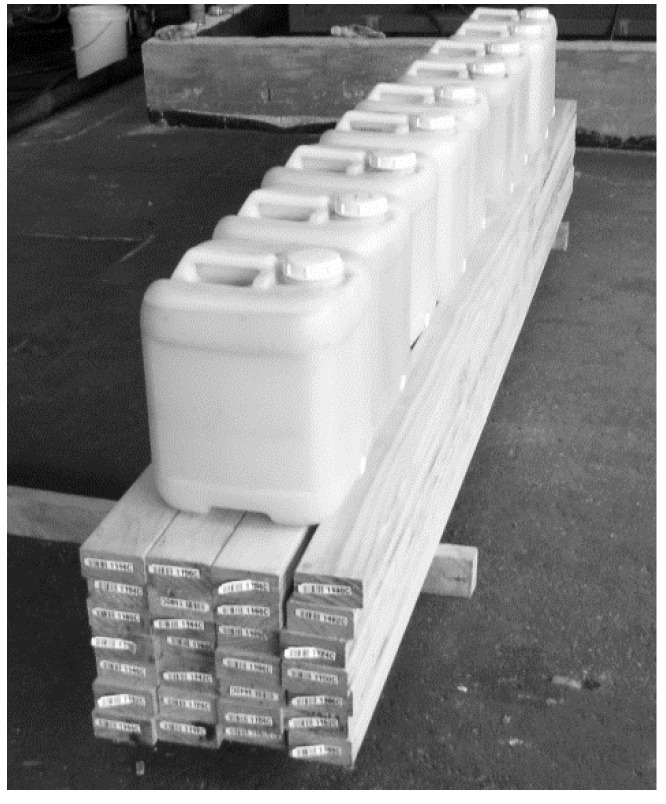
Result of a dewatering experiment using 0.105 m^3^ of green radiata pine timber. The nine 20-litre containers atop the wood stack store the volume of sap recovered from seven carbon dioxide supercritical fluid to gas phase-change cycles. The wood stack was allowed to passively dry over 2 days from 40% moisture content to ambient equilibrium moisture content, <ca. 20%. Photo: R. Franich (13 August 2008).

The step from laboratory experiments towards commercialisation involved testing the dewatering process using industrial-scale wood specimens. This was discussed by Dawson et al. [23] when considering the potential of dewatering using supercritical carbon dioxide for commercial application to green eucalypt wood. To date, the dewatering process has been tested for commercial potential with green radiata pine wood specimens 100 × 50 mm^2^ cross-section and up to 3 m long and using large-diameter pressure vessels and ancillary machinery for generating supercritical carbon dioxide (Pilot plant located at Scion, Rotorua, New Zealand). [23]. Dewatering was successfully accomplished with results the same as those obtained from using the larger laboratory specimens. The scale-up from initial laboratory single small specimens to the 0.105 m^3^ timber stack (Figure 2) was 70,000-fold. In addition to 0.105 m^3^ of boards dewatered to 40% moisture content produced, 180 litres of wood cell sap was recovered. Were this stack of timber conventionally kiln dried, the volume of water atop the wood stack, and approximately 40 litres more water residual in the wood cell walls would be heated, evaporated, and discharged from the kiln exhaust into the air, along with the kiln volatile compounds from heated wood. 

### 2.4. Sap (Cell Water) Chemistry

A significant benefit of the dewatering process described was the lack of any visible kiln-brown-stain when dewatered wood specimens from laboratory experiments were further dried in an oven at temperatures comparable to kiln drying, *viz* 70–120 °C. While green wood dewatering with compressed air using the incubation–decompression process and the carbon dioxide supercritical fluid to gas phase change process produced the same dewatering result with the added benefit of absence of kiln-brown-stain, the latter process had the advantage of greater speed to remove sap. Clearly, the source of chemical precursors of kiln-brown-stain resided in the molecules in the sap, which were removed from the wood by dewatering. 

The composition of green wood sap has been described with the sap samples having been obtained from green wood by water displacement [26] and by dewatering [27]. The wood sap comprised 99% water and 1% solutes, mainly primary metabolites such as the monosaccharides glucose and fructose, the disaccharides sucrose, raffinose, and carbohydrate oligomeric maltodextrins and several amino acids, mainly glutamic acid [26]. Proteins were also identified [26], as were cyclitols such as pinitol, which is the major compound in sap. Sap samples obtained by dewatering also contained lipophilic compounds such as diterpenes [27] owing to their solubility in supercritical carbon dioxide. Many of the sap compounds remained unidentified. 

The sugars and the amino acids identified in the wood cell sap were known to be precursors of Maillard chemical reactions [28]. When heated to temperatures comparable those used in kiln drying, the products of the Maillard reactions form melanoidin dark-brown polymers immediately below the wood surface, which are recognised as kiln-brown-stain. Therefore, removal of the sugars and amino acids from the green wood using dewatering avoided the appearance of kiln-brown-stain and thereby solved one problem related to industrial wood quality where aspects of the surface finish are important.

While the wood sap obtained from the dewatering process would present a disposal problem different from that from any condensate from kiln drying, the presence of the fermentable primary plant metabolites in wood sap could also present an opportunity to use these as feedstock to produce useful products and enable the discharge of the relatively clean water by-product to waste-water treatment.

### 2.5. Magnetic Resonance Imaging (MRI) and Nuclear Magnetic Resonance (NMR) Spectroscopy Studies of the Supercritical Carbon Dioxide to Gas Phase-Change Dewatering Process

In order to gain a deeper insight into the mechanism of the supercritical carbon dioxide dewatering process, experiments using magnetic resonance imaging (MRI) and nuclear magnetic resonance (NMR) spectroscopy were undertaken. To carry out these experiments, a specially designed “autoclave” was designed and constructed from polyether(etherketone) (PEEK), which is an engineering polymer with exceptional strength to withstand the high pressures required to carry out the experiments [29]. To acquire the MRI and NMR data during the dewatering process, it was also necessary to purpose-build an ^1^H and ^13^C doubly-tuned NMR resonator to enable the acquisition of both proton and ^13^C data. This enabled imaging water in the wood specimens, and at the same time, to image carbon (^13^C nuclei) to observe a spatial distribution of carbon dioxide and acquire ^13^C NMR spectra to follow the phase change of carbon dioxide and observe an association of carbon dioxide and wood cell water during the key stages of the process [29]. Since the natural abundance of ^13^C nuclei is only 1%, the carbon dioxide delivered to the “autoclave” was supplemented with 99 atom % ^13^CO_2_ to increase the ^13^C abundance to ca. 10%. This resulted in an improved signal-to-noise ratio, which enabled data acquisition at a rate commensurate with the speed of the dewatering process. 

The ^13^C spectrum for carbon dioxide in the gas phase showed a resonance at 123.0 ppm (relative to tetramethylsilane, 0.0 ppm), which is just slightly downfield from the resonance of supercritical carbon dioxide at 122.7 ppm [30]. The signal assigned to supercritical carbon dioxide dissolved in wood cell water was slightly downfield of this, resulting in an envelope of signals with sufficient resolution to use for ^13^C chemical shift imaging [30]. These experiments showed that during that part of the dewatering process when the pressure of carbon dioxide is increasing, and when the phase changes from compressed gas to supercritical fluid, carbon dioxide not only dissolved in the accessible earlywood cell water at the specimen surface, but it also diffused rapidly through the latewood bands within the tree rings, which being relatively empty of water, served as a conduit to conduct the supercritical carbon dioxide deeply into the wood specimen cross-section, and from there to diffuse into wood cell water of the earlywood within the volume of the specimen. This mechanism of supercritical carbon dioxide diffusion into wood cell water both at all the exposed surfaces and within the wood volume through the latewood bands explains the speed and efficiency of the dewatering process.

From the ^1^H MRI data, the pattern of water loss from the specimen indicated that at the end of the first cycle during the phase change from supercritical fluid to gas, water was expelled from the exposed specimen surfaces, and from the tangential earlywood cells adjacent to the latewood band [31], in a flow direction similar to that for water movement observed during kiln drying [6]. This initial water loss at the first cycle provided a volume of wood cells depleted in cell lumen water for the ingress of a larger volume of supercritical carbon dioxide during the second and subsequent cycles.

Most informative to elucidating the dewatering mechanism was the acquisition of simultaneous proton images, ^13^C spectra, and experiment pressure in situ and in real time using radiata pine green wood specimens contained in the high-pressure autoclave [32], and supplementary ^1^H/^13^C dewatering video cited therein. During the incubation (compression) cycle, the ^13^C spectrum was observed to change shape and intensity from ca. 1 MPa to ca. 7 MPa pressure as carbon dioxide dissolved in cell water. At ca. 10 MPa, the emergence of the signal for supercritical carbon dioxide was observed with change in signal shape, and this signal continued to increase in strength until the maximum applied pressure, 20 MPa, had been attained [32]. On decompression, the supercritical carbon dioxide signal decreased in strength and shape with the reduction of the signal for supercritical carbon dioxide, and at ca. 7 MPa pressure, there were the first indications of water flow from earlywood cells adjacent to the latewood band. The water flow rate increased rapidly when the system pressure was lowered to below 5 MPa concomitant with a reduction in the ^13^C signal intensity [32].

**Figure 3:**
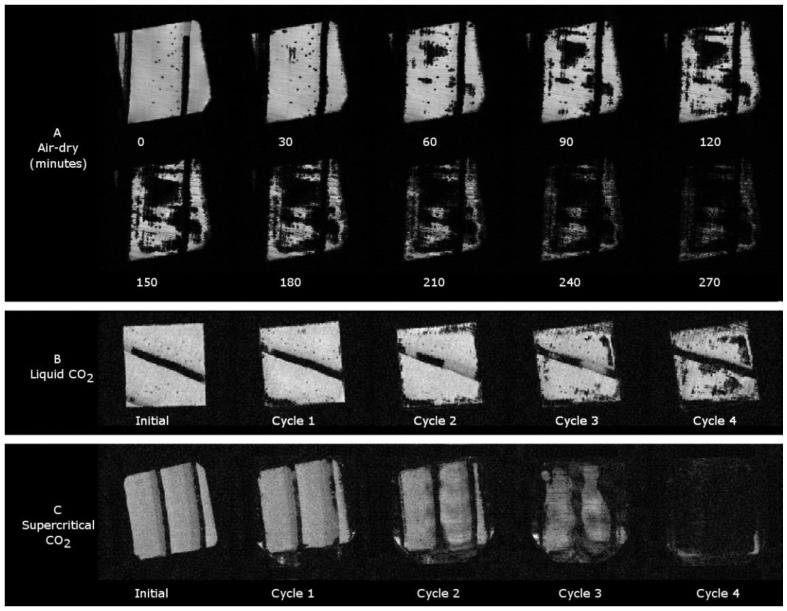
^1^H magnetic resonance images showing radiata pine sapwood specimens undergoing (**A**), conventional drying by water evaporation, and (**B**), cell water displacement by carbon dioxide delivered to the high-pressure “autoclave” by liquid delivery where in the cell at 20 °C, carbon dioxide exists in the gas phase in equilibrium with the liquid phase at 5 MPa, and it was cycled between this pressure and 0.1 MPa, and (**C**), cell water displacement with carbon dioxide undergoing cyclical phase change between supercritical fluid up to 20 MPa, 50 °C, and gas at 0.1 MPa [33] (with permission from de Gruyter).

Proton MRI allowed a comparison of water density change and water movement during conventional air drying; dewatering using pressure cycles of carbon dioxide delivered from liquid supply to the autoclave where, at 20 °C, 5 MPa pressure, would exist in the gas phase in equilibrium with any condensed liquid carbon dioxide; and dewatering using carbon dioxide cycled between supercritical fluid and the gas phase [33]. The ^1^H MR images in Figure 3 show cross-sections of wood specimens, earlywood (light pixels), and latewood (tree-rings, dark pixels) undergoing stages of both drying and dewatering during each experiment.

The ^1^H MR images in Figure 3A show the pattern of water previously described [6] for water movement during conventional air drying, where water evaporating just under the surface of the wood specimen caused water to move from within the earlywood centre towards the surface, creating eventually a drying core with a wet shell until the specimen had dried uniformly throughout the cross-section. 

Dewatering using carbon dioxide gas in equilibrium with liquid phase (from the carbon dioxide liquid delivery cylinder) cycled between 5 and 0.1 MPa showed in the ^1^H MR images a pattern of water density change, as seen in Figure 3B, similar to that for air drying, implying that the mechanisms of air drying and carbon dioxide gas dewatering were similar.

The ^1^H MR images obtained during the dewatering process using carbon dioxide cycled between the supercritical fluid and gas phase, as seen in Figure 3C, showed the pattern of water density change contrasting with the ^1^H MR images obtained during air-drying and from carbon dioxide gas pressure cycling, as seen in Figure 3A,B. Here, water density depletion with each cycle, as seen in Figure 3C, occurred at the periphery of the volume of earlywood, including the boundary with latewood, which is seen especially at the end of cycle three, where it aligns with the dewatering rate data shown in Figure 1a. At the end of the fourth cycle, the wood cell water had been reduced to bound water only, as indicated by the dark image in Figure 3C, which also aligns with the moisture content at the fibre saturation point at the end of the fourth cycle, as shown in Figure 1a.

### 2.6. Predictive Models Developed for Supercritical Carbon Dioxide Dewatering of Green Wood

Complementing the physical chemistry and MRI/NMR experimental results described above are models of the dewatering process which have contributed to the understanding of the dewatering mechanism through the mathematical treatment of mass transfer and diffusion of supercritical dioxide in cell water and the effect of cell porosity and tortuosity of the cell-to-cell pathway for water expulsion [34,35]. The models developed predicted the applied pressure of supercritical carbon dioxide to have the greatest effect on dewatering rate and expelled sap yield compared with the parameters of hold-time at maximum pressure, time for pressure release and phase-change, or carbon dioxide initial temperature. The models derived and physical chemistry results (Figure 1) were in accord, the models providing a means of fine-tuning experimental parameters to assist with the design of plant and processes for dewatering a range of green wood input types. 

Ultimately, the experimental results have shown that it is the features of the anatomy of cell types and their function and cell wall ultrastructure of the bulk wood material of various softwoods and hardwoods that dictate the effectiveness and efficiency of dewatering using the carbon dioxide supercritical fluid-to-gas phase change process. Those features of green radiata pine sapwood make this the ideal material to fit with the process models. 

## 3. Commercialisation of Green Wood Dewatering Using Supercritical Carbon Dioxide

As a process for converting green sawn wood into utility industrial timber for numerous applications, dewatering using carbon dioxide cycled between supercritical fluid and gas phase has some output material quality, processing time, and efflux/environmental advantages, as described above, over conventional wood kiln drying carried out at elevated temperatures. Wood kiln-drying enterprises and kiln standardised operating procedures are well-established, and considerable investment over a long term has already been made into wood drying capability by sawmilling and wood product manufacturing businesses globally. To invest in commercial green wood dewatering enterprise creation would require a clear advantage to be gained over the existing conventional kiln-drying based business models. 

The engineering aspects of the plant and machinery that would be required for a wood dewatering/drying enterprise using supercritical carbon dioxide as the working fluid for green wood dewatering have already been addressed with the construction and successful operation of an impressive enterprise located in Hampen, Denmark [36,37]. There, kiln-dried spruce (*Picea abies* (L.) H. Karst) timber is impregnated with triazole biocides dissolved in supercritical carbon dioxide [38,39]. Spruce timber, which is naturally non bio-durable, after having been conventionally kiln-dried becomes “refractory”, which is a term used to describe wood material that is difficult to treat with water-based biocide formulations and re-dry conventionally: a problem readily solved with the supercritical carbon dioxide biocide delivery process at the Hampen factory.

A potential advantage of applying the green wood dewatering process described above to produce wood material with moisture content at the fibre saturation point and with no resulting distortion, shrinkage, or discolouration is to use the dry wood output from this process as either a finished product in itself (as in the example of eucalypt wood), or as an intermediate towards wood modification or biocide treatment where, as for the triazoles, the modifying agent or biocide is soluble in supercritical carbon dioxide. This feasibility has already been investigated in laboratory experiments, albeit using ethanol-modified supercritical carbon dioxide for the wood-drying step [40]. 

The ability to carry out two key steps in the manufacture of dry, durable wood materials and products at a single site, in one factory where the equipment and machinery could be used for both drying and molecular-modifying steps, would potentially eliminate the multiple handling of wood at intermediate conventional processing steps. An economics analysis comparing conventional wood kiln drying with supercritical carbon dioxide dewatering could bring clarity to the question of commercial viability of the rival process. Apart from the triazole biocides, there are other molecules based on boric acid that would provide protection of wood from decay by fungi and bacteria and also provide protection from attack by wood-boring insects using a single chemical. Trimethyl borate, the simplest boric acid ester, has been used experimentally to deliver boric acid biocide into kiln-dried wood as a hot vapour; this process having been studied using ^11^B NMR and MRI [41]. Trimethyl borate and some boratranes (tricyclic borate esters) [42] are also soluble in supercritical carbon dioxide, making these potential compounds for the modification of dewatered wood using supercritical carbon dioxide as the biocide delivery solvent for the manufacture of biologically durable, quality wood products. 

## 4. Conclusions

Dewatering green sapwood derived from plantation-grown radiata pine and several other softwood and hardwood timber species, using carbon dioxide cycled between the supercritical fluid and gas phase, has proven to be an efficient process for rapidly reducing wood moisture content from as much as 200% (based on dry weight) to 40% (or below, depending on the anatomical structure of the wood). Dewatering has the added benefit of zero volatile emission compared to kiln drying, with all of the sap chemicals being captured in the exudate, which in turn provides a source of numerous chemicals with potential high value to be obtained from them. While the dewatering process has merit for producing dry timber as an industrial product per se, a significant benefit for wood product manufacture may be the ability to sequentially dewater green wood and then undertake wood material modification. For example, biocide molecules dissolved in supercritical carbon dioxide could be introduced in situ, to impart wood product bio-durability without the need to physically handle the wood material. The power of magnetic resonance methods for obtaining in situ proton images of water movement and distribution within the specimen, for obtaining ^13^C spectra of the carbon dioxide phase change during the dewatering process, for ^13^C chemical shift imaging for the distribution of carbon dioxide within the specimen, and simultaneous proton, ^13^C, and pressure data in real time has enabled valuable insights into the dewatering mechanism.
